# Relationships between stress, coping and depressive symptoms among overseas university preparatory Chinese students: a cross-sectional study

**DOI:** 10.1186/1471-2458-11-352

**Published:** 2011-05-19

**Authors:** Pi-Chi Chou, Yu-Mei Y Chao, Hao-Jan Yang, Gwo-Liang Yeh, Tony Szu-Hsien Lee

**Affiliations:** 1 Nursing Department, Taipei Veterans General Hospital, 201, Sec. 2, Shih-Pai Road, Taipei 112, Taiwan; 2 Department of Nursing, College of Medicine, National Taiwan University, 1, Sec 1, Jen-Ai Rd Taipei 100, Taiwan; 3 Department of Public Health, Chung Shan Medical University, 110, Sec.1, Jianguo N. Rd., Taichung City 40201, Taiwan; 4 Department of Health Promotion and Health Education, National Taiwan Normal University, 162, Sec 1, He-Ping East Road, Taipei 10610, Taiwan

## Abstract

**Background:**

Mental health problems in young people are an important public health issue. Students leaving their hometown and family at a young age to pursue better educational opportunities overseas are confronted with life adjustment stress, which in turn affects their mental health and academic performance. This study aimed to examine the relationships among stress, coping strategies, and depressive symptoms using the stress coping framework in overseas Chinese university preparatory students in Taiwan.

**Methods:**

A cross-sectional study was conducted at an overseas Chinese university preparatory institute in Taiwan. Of enrolled overseas Chinese university preparatory students at 2009, 756 completed a structured questionnaire measuring stress, strategies for coping with it, and the Center for Epidemiologic Studies Depression Scale.

**Results:**

High levels of stress significantly predicted the adoption of active, problem-focused coping strategies (*R*^2 ^= 0.13*, p *< .01) and passive, emotion-focused coping strategies (*R*^2 ^= 0.24*, p *< .01). Acceptable CFI, SRMR, and RMSEA values from the structural equation modeling analysis demonstrated that the model satisfactorily fits the stress coping framework, after active coping strategies were eliminated from the model. Results from the Sobel test revealed that passive coping strategies mediated the relation between stress and depressive symptoms (*z *= 8.06, *p *< .001).

**Conclusion:**

Our study results suggested that stress is associated with coping strategies and depressive symptoms and passive strategies mediate the relation between stress and depressive symptoms in overseas Chinese university preparatory students.

## Background

As globalization makes people more mobile, more and more students pursue academics outside their own country. Whether overseas students can successfully adjust to changes in their lives, meet their academic requirements, and maintain their emotional well-being is an important health issue for public health professionals and school counselors. Overseas Chinese students who come from Hong Kong, Macau, Malaysia, etc. leave their home town and family at a young age to pursue better educational opportunities overseas are confronted with an entirely different living environment, education system, and culture when they come to Taiwan. Hence, it is difficult for them to adjust their mindsets and cope with the stresses of everyday life, coursework, and interpersonal relationships [1,2]. In the past 20 years, studies of the factors related to life experiences and depressive symptoms in young people have yielded consistent results, leading researchers to conclude that negative or stressful life events have also been associated with an increased risk of both the onset and recurrence of depression [3].

Previous studies have identified several stressors that overseas Chinese students encounter. They include difficulties with cultural, environmental, interpersonal, emotional, and academic adaptation. Among these, academic adaptation has proved to be the most challenging [4,5], because students have to shoulder the burden of their Chinese parents' high expectations of academic success. It is common for foreign students to also encounter adjustment problems related to lifestyle, mental health, and economic hardships [2]. Among Overseas Chinese students studying in Taiwan, homesickness, fear of failure, depression, and feelings of loneliness were the most frequently reported problems [2].

Granted that mild depression and fear of failure occur, it is still imperative to understand the full extent of mental disturbances among young overseas Chinese students in Taiwan and the factors that cause them. A study conducted by National Taiwan Normal University [6] examined differences in the severity of depressive symptoms among freshmen at the university; 176,026 were Taiwanese students, 1,603 were overseas Chinese students in Taiwan, and 407 non-Chinese students came to Taiwan from other countries, such as America, Canada, Australia, England and Germany, etc. The overseas Chinese students had significantly more severe depressive symptoms than the Taiwanese students and the foreign students. Ying and Lises [7] conducted. a questionnaire study of 171 Chinese students studying in the U.S. and found that those who were mildly depressed before going abroad were the most likely to be depressed while living in the U.S. The authors also found, consistent with the Chinese tradition of saving face, that the international Chinese students refused to seek professional help or did so reluctantly; this reticence increased their depressive symptoms and risk of suicide.

An active, problem-focused coping strategy has been proposed as a way to improve adjustment and academic performance. In a survey of coping strategies among overseas Chinese students, Fang [8] distributed a questionnaire to 527 overseas Chinese university freshmen studying in Taipei. He found that the respondents chose a variety of coping strategies to adjust to emotional, physical and mental stress. Those whose coping strategies were based on a positive attitude and active approach toward dealing with the stress had better outcomes.

The purpose of the present article is to describe the stresses, emotional adjustment problems, coping strategies, and depressive symptoms experienced by overseas Chinese university preparatory students in Taiwan. Specifically, the study examined the relations among stress, adjustment efforts, and depressive symptoms in this population.

## Methods

### Design, participants, and setting

We surveyed overseas Chinese university preparatory students enrolled in a college preparatory program at National Taiwan Normal University in September 2008. The program has three divisions: (1) arts, humanities, law, business, management, and education, (2) physics and engineering, and (3) medicine and agriculture. After one year of study, the students go on to study as freshmen in all departments of all the universities in Taiwan, according to their academic performance and applications.

The questionnaires were anonymous, and the purpose of the research was explained to the respondents. Their confidentiality was assured, and they were informed that they had the right to drop out whenever they wanted. Ethical approval for the survey was obtained from the Institutional Review Board of Taipei Medical University (No. P970119).

### Instruments

The framework for the study was stress coping theory [9]. The questionnaire included three scales: a measure of stress, a measure of corresponding coping strategies, and the Center for Epidemiologic Studies Depression Scale (CES-D) [3,10].

#### Stress scale

The 60-item stress scale was designed by the authors specifically for overseas Chinese university preparatory students. The items were adapted, with permission, from a report by Chang [10], with the wording slightly revised to make the items more relevant to the population sampled. A factor analysis classified the items on four dimensions representing different stress triggers: the pursuit of life goals, academic problems, social problems, and personal/financial problems. A 5-point Likert scale response format was used for all items, anchored at 1 (strongly disagree) and 5 (strongly agree). Higher scores indicate more stress. Cronbach's alpha was 0.74 for life goals, 0.82 for academic problems, 0.83 for social problems, and 0.67 for personal/financial problems.

#### Coping strategies

The 39-item coping strategies was revised from the coping scale from Chang [10] with the wording slightly revised to make the items more relevant to the population sampled. The original coping questionnaire was the Ways of Coping Items [9] that included eight subscales: confrontative coping, distancing, self-control, seeking social support, accepting responsibility, escape/avoidance, planful problem-solving and positive reappraisal [11]. In foundation of the Lazarus and Folkman coping theory, some specialists coordinated and derived the coping strategies scale by factor analysis into approach and avoidant problem/emotion-focused coping [10,12-14]. In this study, the items were adapted, with permission, from a report by Chang [10] that included four subscales: active, problem-focused (seeking support, planning problem solving and accept responsibility, etc.); passive, problem-focused (confrontation, distancing etc.); active, emotion-focused (self control emotion, shift attention, etc.); and passive, emotion-focused (despair, withdraw, etc.) In the questionnaire, each item was assessed using a 5-point Likert scale response format and anchored at 1 (strongly disagree) and 5 (strongly agree). Higher scores indicate the presence of the corresponding coping strategy. In the present study, Cronbach's alpha was 0.77 for active, problem-focused; 0.71 for passive, problem-focused; 0.67 for active, emotion-focused; and 0.66 for passive, emotion-focused.

#### Center for Epidemiologic Studies Depression Scale (CES-D)

The 20-item CES-D has been widely used as a self-report measure of depressive symptoms. For this questionnaire, a 4-point Likert scale response format was used, anchored at 0 (never) and 3 (always). Initial studies suggested that individuals scoring 16 or more on the scale [15] could be used to designate individuals as depressed, however, other cut-off points have been suggested, ranging as high as 21 in the elderly, and 24 to 27 in adolescents [16,17]. In Taiwan, the optimal categorization for teenagers was 18 or below for mild or no depression, 19 to 48 for moderate depression, and 49 or above for severe depression [18]. Because cut-off points are not consistent across studies, in the present study we treated the CES-D scores as continuous (no cut-off points). Cronbach's alpha for the CES-D was 0.89 (*N *= 756).

### Data Analysis

Analyses were performed using SPSS 15.0 and M-plus 5 software. After excluding respondents who declined to participate or returned questionnaires with at least one missing item, 756 were included for the analyses reported in this article. The alpha criterion for statistical significance was set at the level of 0.05.

A general linear model was used to test for the significance of the relationships among the measured variables (stress, coping strategies, depressive symptoms). Structural equation modeling (SEM) using M-plus 5 software was performed to test whether the relationships among the variables conform to an acceptable theoretical framework. The following four indices were frequently employed in the SEM: chi-square test, comparative fit index (CFI), standardized root mean squared residual (SRMR), and root mean square error of approximation (RMSEA) [19]. Because chi-square values are strongly influenced by the number of observations, CFI, SRMR, and RMSEA were given the most weight in assessing the model fit. Finally, the Sobel test was used to test coping strategy as a mediator of the relation between stress and depressive symptoms.

## Results

Of the 944 students who received the survey, 756 (80.08%) completed it. The majority of respondents are from Hong Kong and Macau, altogether accounting for 63.62% (n = 481). Another 190 students (25.13%) are from Malaysia. Students from other countries such as Burma, Japan, Canada and Korea are 85 (11.24%). None of the overseas Chinese respondents are from Mainland China. In this study, 682(90.2%) respondents came to Taiwan less than six months at the time of study interview. The length of stay was not associated with stress, the coping strategy and depressive symptoms.

### Associations between stress and coping strategies

Four types of coping strategies (active, problem-focused; passive, problem-focused; active, emotion-focused; passive, emotion-focused) were regressed on the four aspects of stress (pursuit of life goals, academic problems, social problems, personal/financial problems) by applying general linear modeling to data from our sample of 756 overseas Chinese university preparatory students. Results show that pursuit of life goals (β = -0.19, *p *< .01) and social problems (β = -0.23, *p *< .01) were associated with active, problem-focused coping strategies; the greater the stress related to life goals pursuit or social problems, the less likely respondents were to adopt an active, problem-focused coping strategy (Table 1). Passive, problem-focused strategies were most common among respondents whose stress was associated primarily with pursuit of life goals (β = 0.13, *p *< .01) and personal/financial problems (β = 0.19, *p *< .01.) Likewise active, emotion-focused strategies were positively associated with stress resulting from the pursuit of life goals (β = 0.12, *p *< .05) and personal/financial problems (β = 0.12, *p *< .01), but such strategies were negatively associated with stress from social problems (β = -0.28, *p *< .01). Finally, passive, emotion-focused strategies were positively associated with stress related to the pursuit of life goals (β = 0.17, *p *< .01), social problems (β = 0.27, *p *< .01), and personal/financial problems (β = 0.23, *p *< .01).

**Table 1:** General linear regression analysis of overseas Chinese university preparatory students' stress and coping strategies (*N *= 756)

Coping strategies	Stress	*B*	SE *B*	β*	**Adj. *R***^**2**^
Active, problem-focused coping/ positive					
	Life goals*	-0.18	0.04	-0.19	.09
	Academic problems	-0.02	0.04	-0.02	.04
	Social problems*	-0.22	0.04	-0.23	.10
	Personal/financial problems	0.01	0.04	0.01	.03

Passive, problem-focused coping/negative					
	Life goals*	0.16	0.05	0.13	.04
	Academic problems	-0.05	0.05	-0.04	.02
	Social problems	0.07	0.05	0.06	.03
	Personal/financial problems*	0.22	0.05	0.19	.06

Active, emotion-focused coping/ positive					
	Life goals*	0.13	0.05	0.12	.01
	Academic problems	0.08	0.05	0.08	.01
	Social problems*	-0.30	0.04	-0.28	.02
	Personal/financial problems*	0.12	0.04	0.12	.01

Passive, emotion-focused					
	Life goals*	0.19	0.05	0.17	.12
	Academic problems	-0.07	0.04	-0.06	.06
	Social problems*	0.31	0.04	0.27	.17
	Personal/financial problems*	0.25	0.04	0.23	.14

*p < 0.01

### Associations between coping strategies and depressive symptoms

The results from the general linear model (Table 2) show that the severity of depressive symptoms was negatively associated with active, problem-focused coping strategies (β = -0.16, *p *< .01) and positively associated with passive, emotion-focused coping strategies (β = 0.57, *p *< .01).

**Table 2:** Regression analysis of the effect of overseas Chinese university preparatory students' coping strategies on depressive symptoms (*N *= 756)

Coping strategies	*B*	*SE B*	*β*	***Adjusted R***^**2**^
Active, problem-focused	-3.52	0.67	-0.16*	.03
Passive, problem-focused	-0.61	0.60	-0.03	.05
Active, emotion-focused	0.83	0.68	0.04	.04
Passive, emotion-focused	10.30	0.63	0.57*	.33

*p < 0.01

### Initial structural equation modeling for stress, coping strategies, and depressive symptoms

**Figure 1:**
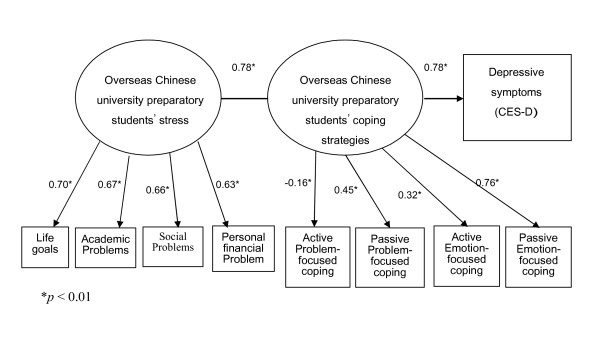
**Initial structural equation modeling for overseas Chinese university preparatory students' stress, coping strategies, and depressive symptoms**.

Results of the initial SEM showed that the four aspects of stress as a group, the four coping strategies as a group, and depressive symptoms each had a significant influence on respondents' coping strategies (Figure 1).

### Model modification

**Figure 2:**
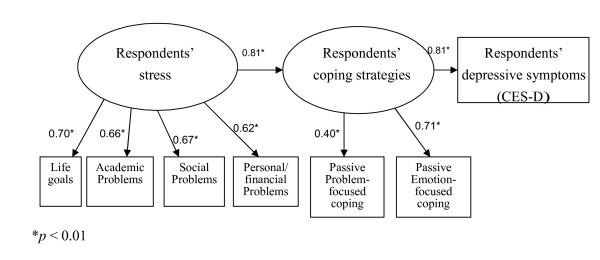
**Revised model for overseas Chinese university preparatory students' stress, coping strategies, and depressive symptoms**.

In the original model (Figure 1), the fit was found to be unsatisfactory according to the CFI, SRMR, and RMSEA indices. The model was thus modified by deleting, one by one, the factors with the lowest loadings. As a result, active, problem-focused strategies (β = -0.16) and negative, emotion-focused strategies (β = 0.32) were removed from the model. After this modification, all the fit indices met the recommended criteria (Table 3). The revised model and the coefficients for each variable are shown in Figure 2.

**Table 3:** Model fit analyses of the stress-and-coping framework using structural equation modeling

Adaptability index	Model 1	Model 2	Model 3
CFI	0.74	0.78	0.90
SRMR	0.10	0.08	0.05
RMSEA	0.16	0.10	0.02

Note. CFI = Comparative Fit Index; SRMR = Standardized Root-mean-square Residual; RMSEA = Root Mean Square Error of Approximation. Suggested criteria for adaptabilityCFI>0.90, 0<SRMR<0.05, RMSEA<0.08

Model 1: Initial structural equation modeling

Model 2: Delete active, problem-focused strategies

Model 3: Delete active, problem-focused and active, emotion-focused strategies

### Mediating effects of negative strategies on the relations between stress and depressive symptoms

**Figure 3:**
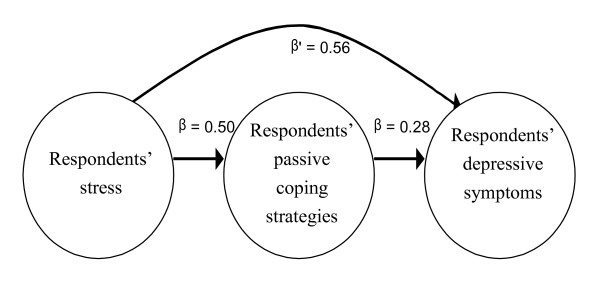
**Relations among overseas Chinese university preparatory students' stress, passive coping strategies, and depressive symptoms**.

To examine the indirect effects of the passive coping strategies on the relations between stress and depressive symptoms, the University of Kansas's online Sobel test was performed (Figure 3). The regression analysis shows that the unstandardized regression coefficient (*B*_1_), representing the association between stress (independent variable) and passive coping strategies (mediator) was 0.838, with a standard error (*SE*_1_) of 0.073. For the association between negative strategies (mediator) and depressive symptoms (dependent variable), *B*_2 _= 21.575 (*SE*_2 _= 1.907). For the association between stress (independent variable) and depressive symptoms (dependent variable), *B*_3 _= 0.70. We then applied the Sobel test formula: *z *= B1*B2/SQRT (*B*2^2^*SE1^2^+*B1*^2^*SE2^2^) = 8.06 (*p *< .01). Because the coefficient *β*', which represents the mediating role of passive strategies vis-à-vis the relation between stress and depressive symptoms, was 0.56, which is less than *β*_3 _(0.70), we conclude that passive strategies mediated the relation between stress and depressive symptoms in our sample [20].

## Discussion

### Stress and coping strategies

The results indicate that academic stress was not directly associated with the use of passive, emotion-focused coping strategies. The other types of stress triggers (pursuit of life goals, social problems, personal/financial problems) were directly related to both active and passive emotion-focused coping strategies. Consistent with previous studies [21,22], we found that our overseas university preparatory Chinese students in Taiwan were more likely to choose passive coping strategies when facing considerable stress, and vice versa. Thus, it can be seen that our respondents preferred passive, emotion-focused to active coping strategies when facing considerable stress.

### Coping strategies and depressive symptoms

We found that the more frequently overseas Chinese pre-undergraduates used active, problem-focused coping strategies, the less severe were the depressive symptoms they reported. Conversely, the more frequently they used passive, emotion-focused strategies, the more severe were the depressive symptoms they reported. When one is confronted with stressful circumstances, adopting a passive strategy to cope with the stress can lead to negative thinking [23], which, in turn, can cause depression [24]. Lee and Chen [25] found that university seniors preferred a passive coping strategy when faced with a high level of stress. On the contrary, when the stress was less acute, they preferred a more active strategy (i.e., seeking emotional and informational social support); as a result, their physical and mental health improved. The results of the present study are consistent with previous studies in that our overseas Chinese university preparatory students' use of passive, emotion-focused strategies significantly associated with the severity of their depressive symptoms.

### Stress, coping strategies, and depressive symptoms

We began by using structural equation modeling to assess the relations among stress, coping strategies, and depressive symptoms in our sample of overseas Chinese university preparatory students. After revising the model, we found that these three variables fit the theoretical framework on which the study was based. In addition, using the Sobel test, this study found that passive strategies tended to mediate the relation between stress and depressive symptoms.

Lazarus and Folkman [9] point out that according to their stress coping theory, coping strategies can help people adjust to their emotions and the stress that activates those emotions, thereby helping them manage the problems caused by the stress. They divided coping strategies into two categories: problem-focused coping and emotion-focused coping. In our study, the respondents surveyed faced many challenges living in an environment (Taiwan) very different from what they had been accustomed to. This was not the case with the local students. The more serious a burden our respondents found the stress resulting from these circumstances to be, the more likely they were to adopt a passive problem-focused or emotion-focused strategy to cope with it. These strategies failed, in that they accompany with depressive symptoms. This result supports the premises of Lazarus and Folkman's [9] stress coping theory (i.e., stress assessment → coping strategies → adaptation): stress, passive coping strategies, and depressive symptoms were closely linked in our sample.

Spence et al. [26] found that poor coping strategies were related to depression. Lai [27] studied the relationship about coping and depressive symptoms with the raw soldiers, it discovered that very depressed people adopted a coping strategy of escape; the more they relied on emotional coping strategies, the more severe their depression. If the participants thought the problem could be solved, they tended to adopt an active, problem-resolving strategy aimed at countering the source of the stress through direct action. If they thought the problem would be hard to solve, they tended to adopt a passive strategy such as escaping, staying way, or temporarily stepping back. Abramson et al. [28] found that an excess of stress can lead to negative emotions and feelings of helplessness and hopelessness. In this respect, our findings are almost identical to those of the studies described earlier in the paragraph.

Overseas Chinese students shoulder their parents' high expectations of academic achievement, based on the traditional norm of cultivating a good reputation. Thus, when the students moved to a new environment (Taiwan), they had to cope with lifestyles as well as economic hardship. These pressures often combined to create extreme stress in our respondents, more so than in the local students.

There were significant correlations among depressive symptoms, stress, and coping strategies in our study. We also found that the adoption of passive coping strategies mediated the relation between stress and depressive symptoms. Although the overseas Chinese students could not choose the kind of stress they had to face, to protect their mental health it was extremely important to let them know the appropriate coping strategy for their particular situation. Health education in schools is potentially one of the most effective ways to reduce and prevent serious health problems, including suicide [29]. Specifically, health education and school counseling programs, as well as those dedicated to overseas students, can regularly hold education workshops or symposia, or even individual consultations, aimed at high-risk students. Such programs could help the overseas Chinese university preparatory students we surveyed, and perhaps others in similar circumstances, to avoid passive coping strategies and give them the support structures they need to pursue more active strategies, even though their family members are many kilometers away. If such programs were put in place, we would expect overseas Chinese students to experience a decrease in depressive symptoms or other affective disturbances through a decrease in the adoption of passive strategies.

## Conclusions

In this study, we analyzed the relations of stress, coping strategies, and depressive symptoms in a sample of overseas Chinese university preparatory students. We found that when these students faced severe stress, they were more likely to associate with passive coping strategies and depressive symptoms. We also found that coping strategies mediated the relation between stress and depressive symptoms. We suggest that in future studies, researchers employ qualitative follow-up interviews of the participants to better understand how they try to cope with the problems they face adapting to life in another country.

## Limitations

There were a few limitations in this study. First, cognitive theories of depression propose that thinking, inferences, attitudes, interpretations, and the way in which they attend to and recall information, can increase their risk of depression [30]. Our study only examined the relationships among stress, coping, and depressive symptoms using the stress coping framework and the results cannot depict the relation of cognitive processes and depressive symptoms amongst an overseas Chinese students in Taiwan. Moreover, personality can play an important role in developing depression. Future study may explore the relation deeper of how respondents perceive, think, behave and interpret academic stress they face while leaving hometown and studying in Taiwan. In addition, given the nature of exclusively self-reported measures, results of this study have to be cautiously interpreted. However, research has shown that self-reported data collected by researchers is generally truthful, reliable and valid of in this kind of population, provided that confidentiality is ensured using anonymous reports and that no sanctions are connected to the answers. Second, our study used a cross-sectional design and causal relations between study variables are not ascertained. A longitudinal approach would be another way to collect more extensive information regarding perceived stress and effects of coping efforts amongst overseas Chinese students which may explain more about causality.

## Conflict of interest statement

The authors declare that they have no competing interests.

## Authors' contributions

PCC draft the manuscript and conducted the study. YMYC and TSHL conceived and planned the study. YMYC, HJY, GLY, and TSHL participated in introduction, results interpretation and discussion of this manuscript. All authors read and approved the final manuscript.

## Pre-publication history

The pre-publication history for this paper can be accessed here:

http://www.biomedcentral.com/1471-2458/11/352/prepub

## References

[B1] Han YF A qualitative study of stress and the coping process for four beginning teachers Master's thesis 2003 National Taiwan Normal University, Taipei, Taiwan21597125

[B2] Huang LH The adjustment problems of Chinese students from overseas J Nurs 1995 3 211 224

[B3] Yang HJ Chiu YJ Soong WT Chen WJ The roles of personality traits and negative life events on the episodes of depressive symptoms in nonreferred adolescents: a 1-year follow-up study J Adolesc Health 2008 42 378 385 10.1016/j.jadohealth.2007.09.017 18346663

[B4] Chou HD College-university-level Overseas Chinese Student Counselors' Concept of Their Profession and Job Satisfaction Master's thesis 2002 National Taiwan Normal University, Taipei, Taiwan21597125

[B5] Lin CW Facing the counseling work and the needs of overseas Chinese university students Chin Guidance Assoc J 1991 27 17 21

[B6] National Taiwan Normal University A physiological and psychology adaptability investigation of the multi-dimensional background of university students Integrated Higher Education Database System in Taiwan 2005 16 1 14

[B7] Ying YW Lises LH Emotional well-being of Taiwan students in the U.S.: an examination of pre- to post-arrival differential Int J Intercult Relat 1991 15 345 366 10.1016/0147-1767(91)90007-4

[B8] Fang H A study on life adjustment of overseas Chinese students at the universities in the Taipei area Master's thesis 1996 Chinese Culture University, Taipei, Taiwan21597125

[B9] Lazarus RS Folkman S Stress, Appraisal and Coping 1984 Springer, New York

[B10] Chang YL A study in adjustment and coping in NTNU freshmen Master's thesis 2001 National Taiwan Normal University, Taipei, Taiwan21597125

[B11] Folkman S Lazarus RS Dunkel-Schetter C DeLongis A Gruen RJ Dynamics of a stressful encounter: cognitive appraisal, coping, and encounter outcomes J Pers Soc Psychol 1986 50 992 1003 3712234 10.1037//0022-3514.50.5.992

[B12] Kao MW A Study of the relationship among self-differentiation, coping strategies, and mental health of high school students Master's thesis 1995 National Taiwan Normal University, Taipei, Taiwan21597125

[B13] Herman-Stabl MA Stemmler M Petersen AC Approach and avoidant coping: implications for adolescent mental health J Youth Adolesc 1995 24 649 665 10.1007/BF01536949

[B14] Pines A Aronson E Career Burnout Causes and Cures 1988 The Free Press, New York

[B15] Radloff LS The CES-D Scale: A self-report depression scale for research in the general population Appl Psychol Meas 1977 1 385 401 10.1177/014662167700100306

[B16] Gotlib IH Lewinsohn PM Seeley JR Symptoms versus a diagnosis of depression: Differences in psychosocial functioning J Consult Clin Psychol 1995 63 90 100 7896995 10.1037//0022-006x.63.1.90

[B17] Roberts RE Lewinsohn PM Seeley JR Screening for adolescent depression: a comparison of depression scales J Am Acad Child Adolesc Psychiatry 1991 30 58 66 10.1097/00004583-199101000-00009 2005065

[B18] Yang HJ Soong WT Kuo PH Chang HL Chen WJ Using the CES-D in a two-phase survey for depressive disorders among nonreferred adolescents in Taipei: a stratum-specific likelihood ratio analysis J Affect Disord 2004 82 419 430 15555693 10.1016/j.jad.2004.04.008

[B19] Chang YH Hsu CM Research Methods and Software Applications 2008 Psychological Publishing Company, Taipei, Taiwan

[B20] Preacher KJ Hayes AF An interactive calculation tool for mediation tests 2009 University of Kansas http://www.people.ku.edu/~preacher/sobel/sobel.htm

[B21] Lee CC Pressure faced and coped with by senior students of National Taiwan Normal University and related factors Master's thesis 2003 National Taiwan Normal University, Taipei, Taiwan21597125

[B22] Wu SC Huang TT Wang W Wei C Psychosocial factors influencing negative thinking: college freshmen J Evid Based Nurs 2008 4 42 50

[B23] Sugiura Y Problem-focused coping strategies and the uncontrollability of thoughts: mediating role of metacognition Japanese J Educ Psychol 2002 50 271 282

[B24] Dyson R Renk K Freshmen adaptation to university life: depressive symptoms, stress, and coping J Clin Psychol 2006 62 1231 1244 10.1002/jclp.20295 16810671

[B25] Lee CC Chen YC Pressure faced and coped with by senior students of National Taiwan Normal University and related factors Chin J Sch Health 2004 44 1 31

[B26] Spence SH Sheffied J Donovan C Problem-solving orientation and attributional style: moderators of the impact of negative life events on the development of depressive symptoms in adolescence? J Clin Child Psychol 2002 31 219 229 10.1207/S15374424JCCP3102_07 12056105

[B27] Lai TJ A study of stress, depression symptoms and coping behaviors in recruited soldiers Master's thesis 2004 National Defense Medical Center, Taipei, Taiwan21597125

[B28] Abramson LY Alloy LB Hankin BL Haeffel GJ MacCoon DG Gibb BE Gotlib IH, Hammen CL (eds) Assessment of depression Handbook of Depression 2002 Guilford, London

[B29] Centers for Disease Control and Prevention Surveillance for characteristics of health education among secondary schools --- school health education profiles (1998) Morbidity and Mortality Weekly Report 2000 49 1 41 10994805

[B30] Gotlib IH Joormann J Cognition and depression: current status and future directions J Clin Psychol 2010 6 285 312 10.1146/annurev.clinpsy.121208.131305 PMC2845726 20192795

